# A prospective cohort study assessing aggressive interventions at the end-of-life among patients with solid metastatic cancer

**DOI:** 10.1186/s12904-022-00970-z

**Published:** 2022-05-16

**Authors:** Chetna Malhotra, Filipinas Bundoc, Isha Chaudhry, Irene Teo, Semra Ozdemir, Eric Finkelstein, on behalf of COMPASS study group, Rebecca A. Dent, Nesaretnam Barr Kumarakulasinghe, Yin Bun Cheung, Rahul Malhotra, Ravindran Kanesvaran, Alethea Chung Pheng Yee, Noreen Chan, Huei Yaw Wu, Soh Mun Chin, Hum Yin Mei Allyn, Grace Meijuan Yang, Patricia Soek Hui Neo, Richard Harding, Lee Lai Heng

**Affiliations:** 1 Lien Centre for Palliative Care, Duke-NUS Medical School, 8 College Road, Singapore, 169857 Singapore; 2 Program in Health Services and Systems Research, Duke-NUS Medical School, 8 College Road, Singapore, 169857 Singapore; 3 National Cancer Centre Singapore, 11 Hospital Dr, Singapore, 169610 Singapore

**Keywords:** End-of-life care, Palliative care, Advanced cancer, Aggressive interventions

## Abstract

**Background:**

Many patients with a solid metastatic cancer are treated aggressively during their last month of life. Using data from a large prospective cohort study of patients with an advanced cancer, we aimed to assess the number and predictors of aggressive interventions during last month of life among patients with solid metastatic cancer and its association with bereaved caregivers’ outcomes.

**Methods:**

We used data of 345 deceased patients from a prospective cohort study of 600 patients. We surveyed patients every 3 months until death for their physical, psychological and functional health, end-of-life care preference and palliative care use. We surveyed their bereaved caregivers 8 weeks after patients’ death regarding their preparedness about patient’s death, regret about patient’s end-of-life care and mood over the last week. Patient data was merged with medical records to assess aggressive interventions received including hospital death and use of anti-cancer treatment, more than 14 days in hospital, more than one hospital admission, more than one emergency room visit and at least one intensive care unit admission, all within the last month of life.

**Results:**

69% of patients received at least one aggressive intervention during last month of life. Patients hospitalized during the last 2–12 months of life, male patients, Buddhist or Taoist, and with breast or respiratory cancer received more aggressive interventions in last month of life. Patients with worse functional health prior to their last month of life received fewer aggressive interventions in last month of life. Bereaved caregivers of patients receiving more aggressive interventions reported feeling less prepared for patients’ death.

**Conclusion:**

Findings suggest that intervening early in the sub-group of patients with history of hospitalization prior to their last month may reduce number of aggressive interventions during last month of life and ultimately positively influence caregivers’ preparedness for death during the bereavement phase.

**Trial registration:**

NCT02850640.

**Supplementary Information:**

The online version contains supplementary material available at 10.1186/s12904-022-00970-z.

## Introduction

Global advancements in cancer treatments have led to more than 50% of patients being treated aggressively [1–6] in some developed countries during their last month of life with 30 to 50% dying in the hospital [1, 2, 4–8]. This is concerning because such care represents low value for the health system, is often inconsistent with patients’ and caregivers’ end-of-life (EOL) preferences and negatively influences caregivers’ perceptions of quality of EOL care received, thereby making adjustment difficult during bereavement [7, 9–14]. Literature has also identified receipt of aggressive interventions as potentially signifying poor quality care [15]. Although difficult to assess at an individual level due to difficulties in prognostication, indicators have been developed to measure it at a population level [15].

Past studies have sought to identify patients vulnerable to receiving aggressive interventions during their last month of life based on their demographics (age, gender and religion), cancer type and pattern of health care use [1, 16–25] using data from either hospital administrative records or retrospective surveys with bereaved caregivers [17, 21, 26–28]. A key concern with the use of administrative data alone is that it cannot provide information about patients’ end of life preference and health status– factors that may influence their choice of care [1, 16, 20, 29, 30]. The validity of retrospective surveys may be limited by caregivers’ knowledge about patients’ experiences and recall bias. Large prospective cohort studies that periodically survey advanced cancer patients until their death and thereafter their bereaved caregivers can help to better assess factors predicting use of aggressive interventions during last month of life and its impact on bereaved caregivers, and such studies are currently lacking.

We conducted a large prospective cohort study of patients with a solid metastatic cancer in Singapore. Our primary aim was to assess the aggressive interventions received by advanced cancer patients during their last month of life and to identify patients at-risk of receiving more aggressive interventions. We assessed the association between number of aggressive interventions received during last month of life and patient demographics (age, gender, socio-economic status, religion), cancer type, pattern of health care use before last month of life (length of hospital stay and palliative care use), health status before last month of life (physical, psychological and functional), and a preference for aggressive care as determined by trade-off between life extension and healthcare cost. In Singapore, a high proportion of health care costs is out-of-pocket [31], therefore, health care costs is an important factor in patient decision-making [32]. We hypothesized that patients who are younger, males, with less education (proxy for low socio-economic status), Buddhists or Taoists, with breast or lung cancer, with longer hospital stay before last month of life, not using a palliative care service before last month of life and with a preference for aggressive care will receive more aggressive interventions during last month of life [19, 33–36] [37–39]. We also hypothesized that patients with a poor physical, psychological or functional health status before their last month of life will receive fewer aggressive interventions during last month of life.

Previous studies suggest that bereaved caregivers of patients receiving more aggressive interventions during last month of life experience difficulties coping [40, 41], rate patients’ care as worse [42, 43], report worse mood [44, 45] and more regret [46, 47] Therefore, as a secondary aim, we assessed the association between number of aggressive interventions received by patients and bereaved caregivers' feelings of low mood, regret and being less prepared for patients’ death. We hypothesize that caregivers of patients receiving more aggressive interventions during last month of life will report being less prepared for patients’ death, are more likely to experience regret with patients’ EOL care and worse mood. These results will provide empirical evidence to develop programs to reduce aggressive interventions during last month of life in efforts to help caregivers prepare emotionally for patients’ death and reduce bereaved caregivers’ distress.

## Materials and methods

### Study design, setting and participants

We used the cohort data of decedents from ‘Cost of Medical Care of Patients with Advanced Serious Illness in Singapore’ (COMPASS) study. COMPASS enrolled 600 patients from outpatient clinics at medical oncology departments of two major hospitals in Singapore between July 2016 and March 2018. Eligibility criteria included diagnosis of stage IV solid malignancy, age > 21 years, Singapore citizenship or permanent residence, cognitively intact (determined through medical records or Abbreviated Mental Test [48] for patients ≥60 years) and Eastern Cooperative Oncology Group [49] performance status ≤2. Patients were surveyed face-to-face every 3 months until death. The study was approved by SingHealth Centralised Institutional Review Board (2015/2781) and National University of Singapore Institutional Review Board (S-20-155). Study details are published [48].

### Study variables

#### Primary outcome

Based on previous literature [15, 28, 50, 51], we assessed the following aggressive interventions - (i) death in the hospital, (ii) use of any anti-cancer treatment (chemotherapy, radiotherapy, hormonal or targeted treatment), (iii) > 14 days in the hospital, (iv) > 1 hospital admission, (v) > 1 Emergency Room (ER) visit and (vi) ≥1 Intensive Care Unit (ICU) admissions, all within the last month of life. We calculated the number of aggressive interventions received by each patient, and used this count as our primary outcome. Each aggressive intervention received equal weight.

#### Secondary outcomes

Eight weeks after patients’ death, we assessed the bereaved caregiver’s feeling of preparedness about patient’s death, regret about patient’s EOL care and mood over the last week. Caregivers rated each of the three on a scale of 0 to 10; a higher score indicated greater feeling of preparedness, greater regret and better mood.

### Independent variables assessed from patients’ last survey answered within the last 2–12 months of life

#### Physical health

We used items from Functional Assessment of Chronic Illness Therapy – Palliative [48, 52] to assess patients’ physical symptoms (pain, breathlessness, constipation, weight loss, vomiting, swelling in body parts, dryness of mouth and throat, lack of energy, nausea and other symptoms; range: 0 to 40); higher score indicated poorer physical health status.

#### Psychological health

We used the Hospital Anxiety and Depression Scale [48, 53, 54], a 14-item scale (range: 0–42) where higher total score represented worse psychological health.

#### Functional health

We used Older American Resources and Services [48, 55] to assess limitations in activities of daily living (ADLs). This is a 7-item scale, assessing patient’s ability (as ‘completely unable to do’ or ‘do with some help’ [= 1], or do without help [= 0]) to eat, dress/undress, take care of own appearance, walk, get in/out of bed, take a bath and use the bathroom. Item scores were summed (range: 0 to 7), a higher score indicated worse functional health.

#### Preference for aggressive care

We asked patients to trade-off life extension with health care cost- “*If you had to make a choice now, would you prefer treatment that extends life as much as possible, or would you want treatment that costs you less?”* Patients responded on scale of 1 to 9, with 1 representing - extend life as much as possible at high cost and 9 representing no life extension at less cost. Responses were classified as maximal life extension [1–4], moderate life extension [5] and no life extension [6–9].

### Other independent variables

#### Length of hospital stay

We used patients’ hospital billing records to assess patients’ cumulative length of hospital stay during the 2 to 12 months before their death.

#### Patient-reported palliative care use

At each survey, we asked patients if they had ever used a palliative or hospice care service (=1 if they reported to have ever used before their last month of life, =0 otherwise).

#### Patient socio-demographics

included age at death, highest education (primary/secondary/above secondary), religion (Free thinking/ Christianity/ Islam/ Buddhist or Taoist/ Hindu or Sikh) and cancer type (breast, respiratory, colorectal, genitourinary or gynaecological and others). We did not use current income as a measure of socio-economic status as it is likely that current income may have declined due to patients’ illness thus may not adequately represent available financial resources.

#### Caregiver socio-demographics

included age, gender and relationship with the patient (spouse/ parent/ child and others).

### Statistical analysis

#### Analysis cohort

We analysed data of patients who died between September 2016 and December 2019, and who answered at least one survey within the last year of their life.

#### Primary analysis

We assessed the association between number of aggressive interventions in last month of life (dependent variable) and our independent variables - patient demographics (age, gender, religion, education, cancer type); pattern of health care use before last month of life (length of hospital stay and palliative care use); health status before last month of life (physical, psychological and functional) and preference for aggressive care. Information on place of death was missing for 14% of the patients. Therefore, as suggested in the literature [56], we first performed a complete case analysis using data from patients with complete information for all aggressive interventions. We then conducted sensitivity analyses using a ‘best’ (assuming all missing observations to have a non-hospital death) and ‘worst’ (assuming all missing observations to have a hospital death) case scenario. For the complete case analyses, we first conducted univariable and then a multivariable analyses using poisson regression. Since the three dimensions of health status were correlated, we estimated three separate multivariable models adding a single dimension of health status as an independent variable in each model (Model 1 - physical, 2 - psychological, 3 - functional). To account for differences in time duration between date of death and date of last patient survey before last month of life, we controlled all models for the time interval between date of survey and date of death (in months).

#### Secondary analysis

We conducted separate multivariable linear regression models using complete case analysis to assess the association between number of aggressive interventions in last month of life (independent variable) and the outcomes – caregivers’ perception of (a) preparedness for patients’ death, (b) regret about patient’s EOL care and (c) mood. To reduce bias, we adjusted for patients’ (age, gender, religion) and caregivers’ (age, gender and relationship with the patient) socio-demographic characteristics. We conducted a sensitivity analyses using the ‘best’ and ‘worst’ case scenario.

All analyses were done using Stata version 16.

## Results

**Fig. 1:**
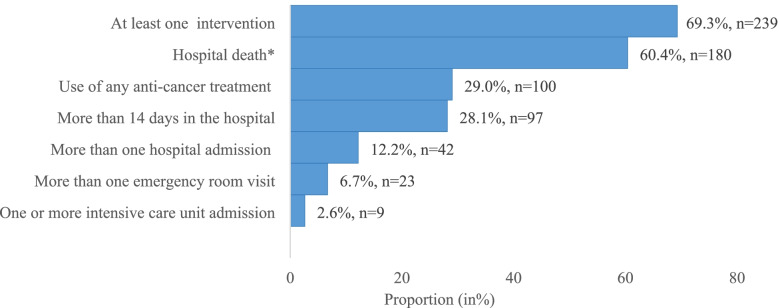
Aggressive interventions received by patients during their last month of life (*N =* 345). * Denominator for proportion of hospital death is 298

Among the 600 patients who participated, 354 (59.0%) died during the study period (Additional file 1: Figure 1). Among them, 345 (97%) patients answered at least one survey during their last year of life and were analysed. Table 1 shows patient demographics, health care use and health status before last month of life. Notably, 67% preferred moderate to maximal life-extension. 127 (37%) caregivers answered the bereavement survey 8 weeks after the patients’ death.

**Table 1:** Characteristics of the deceased patients

	***N =*** **345**
**Baseline Demographics**	
Age at death (in years)	
Mean (SD)	62.4 (10.7)
Median	62.0
Median age and above at death, n (%)	180 (52.2)
Male, n(%)	175 (50.7)
Highest education, n (%)	
Primary or lower	147 (42.6)
Secondary	113 (32.8)
Above secondary	85 (24.6)
Religion, n (%)	
Christian	75 (21.7)
Muslim	60 (17.4)
Buddhist/ Taoist	160 (46.4)
Hindu/ Sikh	10 (2.9)
Free thinker/ No religion	40 (11.6)
Type of cancer, n(%)	
Colorectal	103 (29.9)
Respiratory	98 (28.4)
Genitourinary/Gynaecologic	62 (18.0)
Breast	55 (15.9)
Others^a^	27 (7.8)
**Health service use in the last 2–12 months of life**	
Length of hospital stay, mean (SD)	10.6 (17.1)
Patient-reported palliative care use	87 (25.2)
**Health status and care preferences in the last 2–12 months of life**	
Physical health status (Range: 0–39), mean (SD)	11.3 (8.3)
Functional health status (Range: 0–7), mean (SD)	0.81 (1.80)
Psychological health status (Range: 0–38, mean (SD)	9.2 (7.3)
End of life care preference, n (%)	
Minimal life extension	114 (33.0)
Moderate life extension	146 (42.3)
Maximal life extension	84 (24.3)
Missing information	1 (0.3)

^a^includes head and neck, musculoskeletal, skin and unknown cancer types

Table 2 shows the distribution of bereaved caregiver outcomes.

**Table 2:** Caregiver characteristics at baseline and their bereavement outcomes at 8 weeks after patient’s death

	*N =* 127
	Mean (SD)
**Caregiver characteristics**	
Age	48.2 (14.8)
Male	48 (37.8)
Relationship with patient (Patient is my …), n(%)	
Spouse	54 (42.5)
Parent	53 (41.7)
Others	20 (15.7)
**Bereavement outcomes: Caregiver reported**	
Regret about end-of-life care (Range: 0–10)	3.6 (3.2)
Feeling of preparedness (Range: 0–10)	6.3 (3.0)
Mood in the last week (Range: 0–10)	3.4 (2.6)

Overall, 69% of patients received at least one aggressive intervention during last month of life. Figure 1 shows that 60% (95% CI: 54.6, 66.0) of the patients died in the hospital, 29% (22.3, 32.0) used anti-cancer treatment, 28% (23.4, 33.1) spent > 14 days in the hospital, 12% (8.9, 16.0) had > 1 hospital admission, 7% (3.3, 8.5) had > 1 ER visit, and 3% (0.82, 4.1) had ≥1 ICU admission during their last month of life. 14% of the patients had missing information on their place of death.

Over one-third of the patients (35%) received only aggressive intervention and 18% had three or more aggressive interventions during last month of life.

Table 3 shows the associations between number of aggressive interventions during last month of life and all independent variables for patients with information available on all aggressive interventions (*n =* 298). Univariable analysis showed that patients who had a longer length of hospitalisation within 2–12 months before their death, were Buddhists or Taoists (versus Christian) and with respiratory cancer (versus colorectal cancer) received more aggressive interventions during last month of life. Those with lower functional health before last month of life received fewer aggressive interventions.

**Table 3:** Predictors of number of aggressive interventions received by patients in last month of life

	Univariable analysis^**1,2**^	Multivariable models^**1,3**^		
		Model 1	Model 2	Model 3
	IRR [95% CI]	IRR [95% CI]	IRR [95% CI]	IRR [95% CI]
Physical health status before last month of life	0.99 [0.98, 1.01]	0.99 [0.98, 1.01]		
Psychological health status before last month of life	1.00 [0.98, 1.01]		0.99 [0.98, 1.01]	
Functional health status before last month of life	0.89* [0.83, 0.96]			0.88* [0.81, 0.95]
Length of hospital stay (in days)	1.01* [1.00, 1.01]	1.01* [1.01, 1.02]	1.01* [1.01, 1.02]	1.01* [1.01, 1.02]
Patient-reported palliative care use before last month of life	0.83 [0.66, 1.05]	0.91 [0.71, 1.16]	0.91 [0.70, 1.19]	1.06 [0.82, 1.36]
End of life care preference^3^				
Minimal life extension [Ref]				
Moderate life extension	0.96 [0.77, 1.21]	0.92 [0.72, 1.16]	0.88 [0.69, 1.12]	0.93 [0.74, 1.17]
Aggressive life extension	0.87 [0.67, 1.13]	0.81 [0.62, 1.07]	0.79 [0.59, 1.04]	0.84 [0.64, 1.10]
Age at death above median	0.87 [0.72, 1.05]	0.92 [0.73, 1.14]	0.91 [0.73, 1.15]	0.93 [0.75, 1.16]
Male	1.16 [0.96, 1.41]	1.35* [1.06, 1.71]	1.34* [1.06, 1.70]	1.36* [1.08, 1.72]
Highest education				
Primary or lower [Ref]				
Secondary	1.06 [0.84, 1.33]	1.22 [0.95, 1.57]	1.23 [0.95, 1.59]	1.15 [0.90, 1.48]
Above secondary	1.21 [0.95, 1.53]	1.30 [0.99, 1.71]	1.30 [0.98, 1.71]	1.24 [0.95, 1.63]
Religion				
Christian [Ref]				
Buddhist/ Taoist	1.37* [1.05, 1.78]	1.43* [1.09, 1.89]	1.41* [1.06, 1.89]	1.43* [1.08, 1.88]
Muslim	1.13 [0.82, 1.57]	1.06 [0.75, 1.49]	1.10 [0.77, 1.56]	1.10 [0.78, 1.56]
Hindu/ Sikh	1.22 [0.67, 2.19]	1.20 [0.66, 2.18]	1.19 [0.65, 2.19]	1.38 [0.75, 2.53]
Free thinker/ No religion	0.93 [0.64, 1.35]	0.88 [0.59, 1.29]	0.87 [0.58, 1.31]	0.89 [0.60, 1.31]
Type of cancer				
Colorectal [Ref]				
Breast	1.34 [0.99, 1.81]	1.71* [1.19, 2.45]	1.64* [1.14, 2.37]	1.81* [1.27, 2.55]
Respiratory	1.50* [1.16, 1.94]	1.67* [1.28, 2.18]	1.66* [1.27, 2.18]	1.68* [1.29, 2.19]
Genitourinary/ Gynaecologic	1.17 [0.86, 1.58]	1.28 [0.94, 1.74]	1.25 [0.91, 1.71]	1.29 [0.95, 1.75]
Others	1.23 [0.84, 1.81]	1.45 [0.97, 2.19]	1.32 [0.85, 2.05]	1.40 [0.94, 2.11]
Number of months before death survey was answered	0.99 [0.93, 1.05]	1.00 [0.94, 1.06]	1.00 [0.94, 1.06]	0.98 [0.92, 1.05]

^1^Poisson regression; ^2^*n =* 298^; 3^*n =* 297; * Statistically significant at the 5% level

Multivariable analysis showed that patients who had a longer length of hospitalisation within the last 2–12 months before their death, males, Buddhists or Taoists (versus Christian) and with breast or respiratory cancer (versus colorectal cancer) (all models) received more aggressive interventions. On the contrary, patients with worse functional (model 3) health before their last month of received fewer aggressive interventions. No significant association was found between number of aggressive interventions during last month of life and other independent variables. (Table 3).

The results from our sensitivity analyses (*n =* 345) (Additional file 1: Table 1) regarding the association between number of aggressive interventions during last month of life and functional health, length of hospitalization, type of cancer, religion and gender were consistent with those from the complete case analysis. We also found that patients with worse physical health (model 1) received fewer aggressive interventions in the last month of life in the ‘best’ case scenario.

Patients of 119 out of 127 bereaved caregivers had complete data on number of aggressive interventions during last month of life. Table 4 shows that 8 weeks after patients’ death, bereaved caregivers of patients who received more aggressive interventions felt less prepared for patients’ death. Contrary to our hypothesis, we did not find patients’ receipt of aggressive interventions during last month of life to be associated with bereaved caregivers’ regret about EOL care and their mood.

**Table 4:** Association between number of aggressive interventions during last month of life and bereaved caregiver outcomes^a^ (*N =* 119)

	Caregiver regret about end-of-life care	Caregiver feeling of preparedness	Caregiver mood in the last week
	Coef. [95% CI]	Coef. [95% CI]	Coef. [95% CI]
Number of aggressive interventions in last month of life	0.15 [−0.35, 0.64]	−0.50* [−0.98, −0.02]	−0.37 [−0.78, 0.05]

* Statistically significant at the 5% level

Estimates adjusted for for patient’s age, gender and religion and caregiver’s age, gender and relationship with patient

The association between patients’ receipt of aggressive interventions during last month of life and bereaved caregivers’ lack of preparedness persisted in the sensitivity analyses using ‘best’ and ‘worst’ case scenarios. However, both the ‘best’ and ‘worst’ case scenarios also showed that caregivers of patients with more aggressive interventions experienced worse mood during the bereavement (Additional file 1: Table 2).

## Discussion

Using data from a prospective cohort study, we assessed the number of aggressive interventions in last month of life received by patients with a solid metastatic cancer in Singapore. We found that 69% of the patients in our sample received at least one aggressive intervention during last month of life. This is comparable to estimates from other developed countries [1–4, 7, 8]. The proportion of patients dying in hospital was however higher compared to other settings (Additional file 1: Table 3). This may be due to low rates of nursing home admissions in Singapore [57, 58] or because the public health insurance (Medshield) in the country subsidizes cost for hospitalizations but not in-patient hospice admissions [59, 60].

Past studies show that many advanced cancer patients experience repeated hospitalizations even before their last month of life [59, 61]. Our results show that patients hospitalized during the last 2–12 months of their life were at high risk of receiving more aggressive interventions during last month of life [62]. If providers can ex-ante identify hospitalized patients at-risk of dying within the next 1 year (e.g. through surprise question, other prognostic indicators), they may institute measures to prevent these patients from being treated aggressively during last month of life [63]. This may include initiating goals of care discussions with them that is known to improve patients’ prognostic understanding, reduces use of aggressive interventions and increases hospice use [36, 64–67].

Consistent with previous research [16, 21–23], our results show that patients with breast or respiratory cancer and male patients received more aggressive interventions during last month of life. We, however, did not find a significant association between education level and number of aggressive interventions during last month of life. Similarly, no significant association was observed between palliative care use before the last month of life with number of aggressive interventions during last month of life. This might be because of the possible mediating effect of other independent variables in our model. We were unable to test this mediating effect as the exact time of initiation of use of palliative care service was not captured in the survey. This can be a topic of future research.

We also found that Buddhist or Taoist patients received more aggressive interventions during last month of life. Although both Buddhist and Taoist teachings emphasize the inevitability of death, some Taoists believe that death may lead to an afterlife torture and suffering, and therefore they may prefer more aggressive interventions to extend life [25].

Our results showed that patients with worse functional health before their last month of life received fewer aggressive interventions during last month of life. Poor health is known to be associated with a more realistic understanding of prognosis [16, 18, 29]. Past studies show that patients with a more realistic understanding of prognosis are more likely to prefer and be recommended by their health care providers to use comfort care than aggressive interventions [11, 67–69], and are more likely to be referred to a palliative care service [70].

Our primary and sensitivity analyses showed that bereaved caregivers of patients receiving more aggressive interventions during their last month of life reported being less prepared for patient’s death. This is consistent with previous studies [40, 44]. Our sensitivity analysis further highlighted that bereaved caregivers of patients receiving more aggressive interventions during their last month of life experienced worse mood. Past studies have also similarly suggested that bereaved caregivers of patients receiving aggressive care during last month of life experience difficulties coping and even major depression [40, 71]. However, in contrast to our hypothesis, we did not find any association between patients’ receipt of aggressive interventions and their caregivers’ feelings of regret. This may be because many caregivers may be choosing aggressive interventions for patients in order to avoid experiencing regret [72, 73]. Overall, findings suggest that when patients are treated aggressively during their last month of life, their caregivers are affected adversely, and thus require support during the course of patient’s treatment and during bereavement. Clinicians should also involve caregivers in EOL discussions and discuss pros and cons of each intervention with them so that they can weigh in on any potential perceived benefit of the intervention against any harm, and also be more prepared for patient’s death [40, 74].

Our main strength is the use of prospective longitudinal data merged with administrative data to provide a comprehensive account of patients’ EOL care and experience. However, the study also had limitations. First, data on use of palliative care service was self-reported by patients and not available regarding its time of initiation, duration and type (i.e. whether hospital, home or hospice based). We also did not have information on whether the patient had goals of care discussions. Second, we were unable to access information on place of death for some patients in one of the participating hospitals, resulting in this information being missing for 14% of the patients. Third, data on bereaved caregivers was available only for a sub-sample of deceased patients. Fourth, similar to other studies relying on administrative data to assess aggressive interventions [19, 23], we were unable to identify whether chemotherapy and other treatments used during the last month of patients’ life were used as an active treatment or with a palliative intent, and thus may have over-estimated the use of aggressive interventions during last month of life. Fifth, we retrospectively defined aggressive interventions after patients’ death. It is more difficult to identify it prospectively in clinical practice, especially for the sub-group of patients experiencing rapid deterioration due to cancer or its treatments. Sixth, we were unable to assess each aggressive intervention in isolation due to small sample size and low prevalence of certain interventions in the last month of life before death (≥1 ICU admission (*n =* 9; 2.6%) and > 1 ER admission (*n =* 23; 6.7%)). Seventh, we weighted each intervention equally. A similar approach has been used in previous studies [7, 37, 64]. However, patients and other stakeholders such as caregivers and physicians, may assign different weights to each aggressive intervention. Assessing appropriate weights for each intervention should be a topic of future research. Eighth, time from diagnosis may have influenced patient’s uptake of aggressive care, however, we did not have access to this data. Lastly, caregivers’ preferences for patients’ care may have influenced patients’ use of aggressive interventions during their last month of life. Assessing this could be a topic of future research.

## Conclusions

We found that a high proportion of patients dying with a solid metastatic cancer in Singapore were treated aggressively in their last month of life. Patients with history of hospitalization prior to their last month were at a greater risk of receiving more aggressive interventions during their last month of life. Findings suggest that intervening early in the sub-group of patients with history of hospitalization prior to their last month may reduce number of aggressive interventions during last month of life and ultimately positively influence caregivers’ preparedness for death during the bereavement phase.

## Supplementary Information

**Additional file 1.**

## Data Availability

The deidentified datasets generated during and/or analysed during the current study are not publicly available but are available from the corresponding author on reasonable request. Every request will be reviewed by the approving Institutional Review Board and the researcher will need to sign a data access agreement with National University of Singapore after approval.
