# Supplementary material for: A prospective cohort study assessing aggressive interventions at the end-of-life among patients with solid metastatic cancer
Source: BMC Palliat Care. 2022 May 16;21:73. doi: 10.1186/s12904-022-00970-z (PMC9109395; doi:10.1186/s12904-022-00970-z)
Supplement: Supplementary file 1 — Additional file 1. [file 12904_2022_970_MOESM1_ESM.docx]

**ADDITIONAL FILE**

**Figure 1. COMPASS participant flow diagram**

Excluded (n=2)

- Withdrew consent before completion of baseline (n=2)

Approached (n=1,137)

Excluded (n=95)

- Did not meet inclusion criteria (n=95)

Eligible (n=1,042)

Declined to participate (n=393)

- Not interested/ Indecisive (n=306)
- No time to participate (n=4)
- Research fatigue (n=20)
- Too ill or has hearing, vision, or speech impairment (n=34)
- Caregiver rejected (n=29)

Enrolled (n=649)

Baseline Data Collected (n=647)

- Records review only (n= 47)
- Survey and records review (n= 600)

Deceased during the study period (n=354)

- Excluded from analysis (n=9)

Did not answer the survey in last year of life

**Analysed (n=345)**

Excluded (n=246)

- Patients still alive (n=246)

**Table 1. Sensitivity analysis of predictors of number aggressive interventions received by patients in last month of life**

|  | **Best-case scenario^1,2^**  **(assuming all missing data for place of death as non-hospital deaths)** | | | **Worst-case scenario^1,2^**  **(assuming missing data for place of death as hospital deaths)** | | |
| --- | --- | --- | --- | --- | --- | --- |
|  | **Model 1** | **Model2** | **Model 3** | **Model 3** | **Model 2** | **Model 3** |
|  | **IRR [95% CI]** | **IRR [95% CI]** | **IRR [95% CI]** | **IRR [95% CI]** | **IRR [95% CI]** | **IRR [95% CI]** |
| Physical health status before last month of life | 0.99* [0.97, 1.00] |  |  | 1.00 [0.98, 1.01] |  |  |
| Psychological health status before last month of life |  | 0.99 [0.97, 1.00] |  |  | 0.99 [0.98, 1.01] |  |
| Functional health status before last month of life |  |  | 0.87* [0.81, 0.94] |  |  | 0.89* [0.83, 0.96] |
| Length of hospital stay | 1.01* [1.01, 1.02] | 1.01* [1.01, 1.02] | 1.01* [1.01, 1.02] | 1.01* [1.01, 1.02] | 1.01* [1.01, 1.02] | 1.01* [1.01, 1.02] |
| Patient-reported palliative care use before last month of life | 0.95 [0.74, 1.21] | 1.01 [0.78, 1.31] | 1.09 [0.85, 1.40] | 0.89 [0.71, 1.12] | 0.94 [0.73, 1.20] | 1.05 [0.83, 1.33] |
| End of life care preference^3^ |  |  |  |  |  |  |
| Minimal life extension [Ref] |  |  |  |  |  |  |
| Moderate life extension | 0.97 [0.77, 1.21] | 0.90 [0.72, 1.13] | 1.01 [0.82, 1.40] | 0.90 [0.72, 1.11] | 0.86 [0.69, 1.06] | 0.90 [0.73, 1.10] |
| Aggressive life extension | 0.92 [0.71, 1.20] | 0.87 [0.66, 1.14] | 0.98 [0.76, 1.27] | 0.84 [0.66, 1.09] | 0.81 [0.63, 1.06] | 0.86 [0.67, 1.10] |
| Age at death above median | 0.89 [0.73, 1.10] | 0.88 [0.71, 1.09] | 0.91 [0.74, 1.12] | 0.89 [0.73, 1.09] | 0.89 [0.72, 1.09] | 0.90 [0.74, 1.10] |
| Male | 1.28* [1.02, 1.61] | 1.33* [1.06, 1.67] | 1.33* [1.06, 1.66] | 1.36* [1.09, 1.68] | 1.35* [1.08, 1.68] | 1.36* [1.10, 1.69] |
| Highest education |  |  |  |  |  |  |
| Primary or lower [Ref] |  |  |  |  |  |  |
| Secondary | 1.13 [0.89, 1.42] | 1.13 [0.88, 1.44] | 1.07 [0.84, 1.35] | 1.15 [0.92, 1.44] | 1.16 [0.92, 1.46] | 1.11 [0.89, 1.39] |
| Above secondary | 1.28 [0.99, 1.66] | 1.24 [0.96, 1.62] | 1.19 [0.92, 1.53] | 1.22 [0.96, 1.57] | 1.22 [0.95, 1.58] | 1.19 [0.93, 1.52] |
| Religion |  |  |  |  |  |  |
| Christian [Ref] |  |  |  |  |  |  |
| Buddhisht/ Taoist | 1.42* [1.09, 1.85] | 1.38* [1.05, 1.83] | 1.43* [1.10, 1.87] | 1.36* [1.06, 1.74] | 1.35* [1.03, 1.75] | 1.36* [1.06, 1.74] |
| Muslim | 1.17 [0.84, 1.62] | 1.22 [0.87, 1.72] | 1.24 [0.89, 1.72] | 1.11 [0.81, 1.51] | 1.16 [0.83, 1.61] | 1.15 [0.84, 1.57] |
| Hindu/ Sikh | 1.24 [0.68, 2.25] | 1.20 [0.65, 2.19] | 1.42 [0.78, 2.58] | 1.14 [0.64, 2.01] | 1.12 [0.63, 2.00] | 1.26 [0.71, 2.25] |
| Free thinker/ No religion | 0.97 [0.66, 1.42] | 0.98 [0.66, 1.46] | 1.00 [0.68, 1.46] | 0.85 [0.59, 1.23] | 0.85 [0.57, 1.24] | 0.86 [0.59, 1.24] |
| Type of cancer |  |  |  |  |  |  |
| Colorectal [Ref] |  |  |  |  |  |  |
| Breast | 1.61* [1.14, 2.26] | 1.58* [1.12, 2.24] | 1.76* [1.26, 2.46] | 1.62 * [1.17, 2.24] | 1.54* [1.10, 2.15] | 1.68* [1.22, 2.31] |
| Respiratory | 1.53* [1.19, 1.97] | 1,50* [1.16, 1.94] | 1.57* [1.22, 2.03] | 1,51 [0.90, 1.60] | 1.49* [1.17, 1.90] | 1.53* [1.21, 1.94] |
| Genitourinary/ Gynaecologic | 1.27 [0.94, 1.71] | 1,25 [0.92, 1.69] | 1.28 [0.95, 1.72] | 1.20 [0.90, 1.60] | 1.17 [0.87, 1.56] | 1.22 [0.92, 1.62] |
| Others | 1.41 [0.95, 2.09] | 1.24 [0.82, 1.90] | 1.39 [0.94, 2.06] | 1.30 [0.88, 1.90] | 1.17 [0.78, 1.78] | 1.26 [0.86, 1.84] |
| Number of months before death survey was answered | 0.98 [0.92, 1.04] | 0.98 [0.92, 1.04] | 0.97 [0.91, 1.03] | 0.99 [0.94, 1.05] | 0.99 [0.93, 1.05] | 0.98 [0.92, 1.04] |

^1^Poisson regression; ^2^n=344; * Statistically significant at the 5% level

**Table 2. Sensitivity analysis of the association between number of aggressive interventions in last month of life and bereaved caregiver outcomes (N=127)**

|  | **Best-case scenario**  **(assuming all missing data for place of death as non-hospital deaths)** | | | **Worst-case scenario**  **(assuming missing data for place of death as hospital deaths)** | | |
| --- | --- | --- | --- | --- | --- | --- |
|  | Caregiver regret about end-of-life care | Caregiver feeling of preparedness | Caregiver mood in the last week | Caregiver regret about end-of-life care | Caregiver feeling of preparedness | Caregiver mood in the last week |
|  | Coef. [95% CI] | Coef. [95% CI] | Coef. [95% CI] | Coef. [95% CI] | Coef. [95% CI] | Coef. [95% CI] |
| Number of aggressive interventions in last month of life | 0.20 [-0.28, 0.69] | -0.58* [-1.05, -0.12] | -0.41* [-0.81, -0.01] | 0.15 [-0.32, 0.63] | -0.59* [-1.04, -0.13] | -0.46* [-0.85, -0.07] |
| Patient's age at death above median | 0.81 [-0.53, 2.15] | -0.03 [-1.32, 1.27] | 0.74 [-0.37, 1.85] | 0.82 [-0.53, 2.16] | -0.04 [-1.33, 1.26] | 0.73 [-0.37, 1.83] |
| Male patient | -0.64 [-1.88, 0.60] | 0.16 [-1.04, 1.36] | 0.61 [-0.42, 1.64] | -0.62 [-1.87, 0.62] | 0.14 [-1.05, 1.34] | 0.61 [-0.41, 1.63] |
| Patient's religion |  |  |  |  |  |  |
| Christian [Ref] |  |  |  |  |  |  |
| Buddhist/ Taoist | -0.75 [-2.15, 0.66] | 1.39* [0.03, 2.75] | -0.58 [-1.75, 0.59] | -0.72 [-2.13, 0.69] | 1.39* [0.03, 2.75] | -0.55 [-1.71, 0.60] |
| Muslim | -1.36 [-3.08, 0.36] | 2.11* [0.45, 3.77] | 0.00 [-1.42, 1.43] | -1.35 [-3.08, 0.38] | 2.15* [0.48, 3.81] | 0.05 [-1.37, 1.47] |
| Hindu/ Sikh | 3.05 [-0.21, 6.32] | -0.80 [-3.95, 2.36] | -1.68 [-4.39, 1.02] | 3.11 [-0.15, 6.38] | -0.82 [-3.96, 2.33] | -1.65 [-4.34, 1.03] |
| Free thinker/ No religion | 0.29 [-1.62, 2.19] | 0.40 [-1.44, 2.24] | 0.24 [-1.33, 1.82] | 0.30 [-1.61, 2.20] | 0.37 [-1.46, 2.21] | -.23 [-1.34, 1.79] |
| Caregiver's age | -0.10* [-0.16, -0.03] | 0.05 [-0.01, 0.11] | -0.03 [-0.08, 0.02] | -0.10* [-0.16, -0.03] | 0.05 [-0.01, 0.11] | -0.03 [-0.08, 0.02] |
| Male Caregiver | -0.54 [-1.80, 0.71] | 0.08 [-1.13, 1.29] | -0.68 [-1.72, 0.36] | -0.54 [-1.79, 0.72] | 0.04 [-1.17, 1.26] | -0.71 [-1.74, 0.33] |
| Caregiver's relationship with patient (Patient is…) |  |  |  |  |  |  |
| Spouse [Ref] |  |  |  |  |  |  |
| Parent | -0.81 [-2.82, 1.20] | 0.49 [-1.46, 2.44] | -0.62 [-2.29, 1.05] | -0.84 [-2.85, 1.18] | 0.50 [-1.44, 2.44] | -0.64 [-2.30, 1.02] |
| Others | 0.01 [-1.67, 1.70] | -0.19 [-1.81, 1.44] | -0.04 [-1.43, 1.35] | -0.01 [-1.69, 1.67] | -0.19 [-1.82, 1.43] | -0.07 [-1.45, 1.32] |

* Statistically significant at the 5% level

**Table 3: Findings from published studies on aggressive intervention in last month of life**

| **Study** | **Country** | **Region and year(s) of sample collection** | **Cancer type** | **Hospital death (%)** | **Use of any anti-cancer treatment (%)** | **More than 14 days in the hospital (%)** | **More than one hospital admission (%)** | **More than one emergency room visit (%)** | **One or more intensive care unit admission (%)** |
| --- | --- | --- | --- | --- | --- | --- | --- | --- | --- |
| Hu, Yasui et al. (2014) | Canada | Alberta, 2006-2009 | Colorectal | 50.1 | — | — | 9.5 | 12.5 | 2.2 |
| Hui, Kim et al. (2014) | U.S.A | Houston, 2009-2010 | All | 27 | 26 | 14 | 17 | 19 | 10 |
| Cheung, Earle et al. (2015) | Canada | Ontario, 2005-2009 | All | — | — | — | 6.3 | 14.7 | 5.5 |
| Kao and Chiang (2015) | Taiwan | All cities, 2000-2011 | Liver | 48 | 11.3 | — | 22.5 | 4.9 | 18.8 |
| Falchook, Dusetzina et al. (2017) | U.S.A | 14 states, 2007-2014 | Lung and bronchus, colon and rectum, breast (female only), pancreas, prostate | 30.3–35.4 | — | — | — | 1.5-2.2 | 15.9-20.6 |
| Presley, Han et al. (2020) | U.S.A | All states, 2012 | Lung | — | — | — | 3.6 | — | 9.8 |
